# Case Report: Locking Plate for Cubitus Varus Correction in a 7-Year-Old Girl With Osteogenesis Imperfecta

**DOI:** 10.3389/fped.2021.781703

**Published:** 2022-01-12

**Authors:** Pan Hong, Ruikang Liu, Saroj Rai, Jin Li

**Affiliations:** ^1^Department of Orthopaedic Surgery, Union Hospital, Tongji Medical College, Huazhong University of Science and Technology, Wuhan, China; ^2^First School of Clinical Medicine, Tongji Medical College, Huazhong University of Science and Technology, Wuhan, China; ^3^Department of Orthopaedics and Trauma Surgery, Blue Cross Hospital, Kathmandu, Nepal

**Keywords:** cubitus varus, osteogenesis imperfecta, locking plate, case report, pediatrics

## Abstract

**Background:** Cubitus varus deformity is a common complication of untreated elbow fractures in children. However, cubitus varus in osteogenesis imperfecta (OI) children is a rare but challenging situation. To the author's knowledge, this is the first study discussing the correction of cubitus varus deformity in patient with OI.

**Case Presentation:** Here we report a case of a 7-year-old OI girl with cubitus varus deformity due to a supracondylar fracture of humerus 3 year ago. The patient's parent gave a history of supracondylar fracture of left humerus in 2015. Without medical intervention, the patient was admitted into our institution for corrective surgery with the diagnosis of osteogenesis imperfecta and cubitus varus deformity in the left arm.

**Result:** Medications including calcium, vitamin D and bisphosphonates were administered before the corrective surgery of cubitus varus, and a single locking plate was used to fixate the osteotomy. After the surgery, the appearance and range of motion (ROM) of the left arm was almost normal. Combined with gradual rehabilitation, the ROM of the left arm was normal without pain during daily use within the 1-year follow up. The hardware was removed as the nailing of the forearm fractures was performed at the same time. In the latest follow-up in September 2021, the appearance and ROM of the left arm was normal.

**Conclusion:** Cubitus varus is a common deformity in children with elbow injuries, but it presents a challenging situation in compound fractures in OI patients. Locking plate combined with meticulous pharmacological intervention provides a good option for corrective surgery of cubitus varus in patients with OI.

## Background

Cubitus varus deformity is a common complication of elbow fractures in children (1). The surgical interventions for this deformity remain controversial (2). Many techniques have been reported to correct this deformity to avoid long-term complications, including tardy ulnar nerve palsy (3), post-erolateral rotatory instability (4), and secondary distal humeral fracture (5). However, reports on the correction of cubitus varus deformity in patients with osteogenesis imperfecta (OI) patients remain sparse.

Osteogenesis imperfecta (OI), also known as “brittle bone disease,” is a heritable collagen-related disorder characterized by abnormal or reduced production of collagen (6). Mutations in the genes COLIA and COL1A2 that codify for the type 1 collagen chains and other genes that function within the collagen biosynthesis pathway are involved in mechanisms of OI (7, 8). Patients with OI frequently manifest unintentional fracture, growth retardation, and deformity due to bone fragility (9). Besides skeletal features, other symptoms like dental and craniofacial abnormalities, muscle weakness, hearing loss, respiratory, and cardiovascular complications are also reported in patients with OI (8). OI occurs in about 1 in 10,000–20,000 live births, but the incidence might be higher because of its heterogeneity (10–12).

Due to poor bone quality, the treatment of supracondylar fractures in OI is quite difficult, and the treatment of post-traumatic deformity is even more challenging. Here, we presented a case of cubitus varus deformity in a 7-year-old girl with OI. Her elbow deformity was successfully treated by closing-wedge osteotomy fixated by a locking plate. Eighteen months after the removal of internal fixation, the patient demonstrated satisfactory recovery.

## Case Presentation

**Figure 1:**
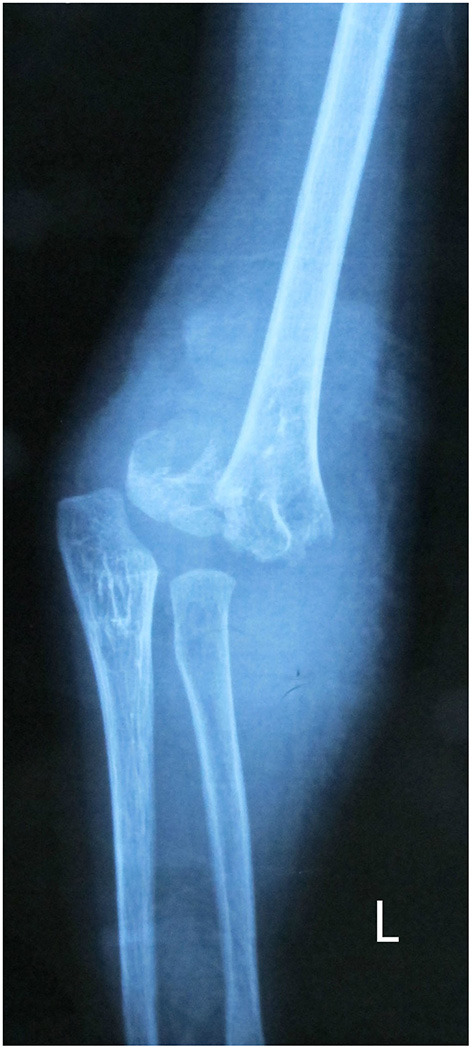
Gartland type III supracondylar fracture of a 4-year-old girl.

A 7-year-old girl with bilateral femoral deformity and fresh bilateral femoral shaft fracture was presented in our clinic. The patients appeared short stature (112 cm height) and very low BMI (13.6 kg/m^2^). During her early childhood, the patient frequently sustained multiple fractures on the lower extremities by trivial trauma. Initially, she was suspected of having OI at a municipal hospital. However, no medical therapy was administered. On physical examination, the patient displayed 30 degrees cubitus varus with a history of supracondylar humeral fracture (SCHF) 3 years ago (see Figure 1). The patient received closed reduction and external fixation for a Gartland type III SCHF when she was 4 years old. However, the diagnosis of OI was still not mentioned in the discharge paper after the first surgery. No supplemental drugs, including calcium, vitamin D, and bisphosphonates, were given to this patient to increase her bone mass after the SCHF surgery. Fortunately, according to her parents, no elbow fractures occurred afterward. Normal curvature of the spine existed at the physical examination without previous history of spine fracture. No symptoms manifested in auditory and dental health. OI diagnosis was confirmed by genetic testing as COL1A2, P3H1, and PPIB were all positive. However, the patient had no family history of OI.

**Figure 2:**
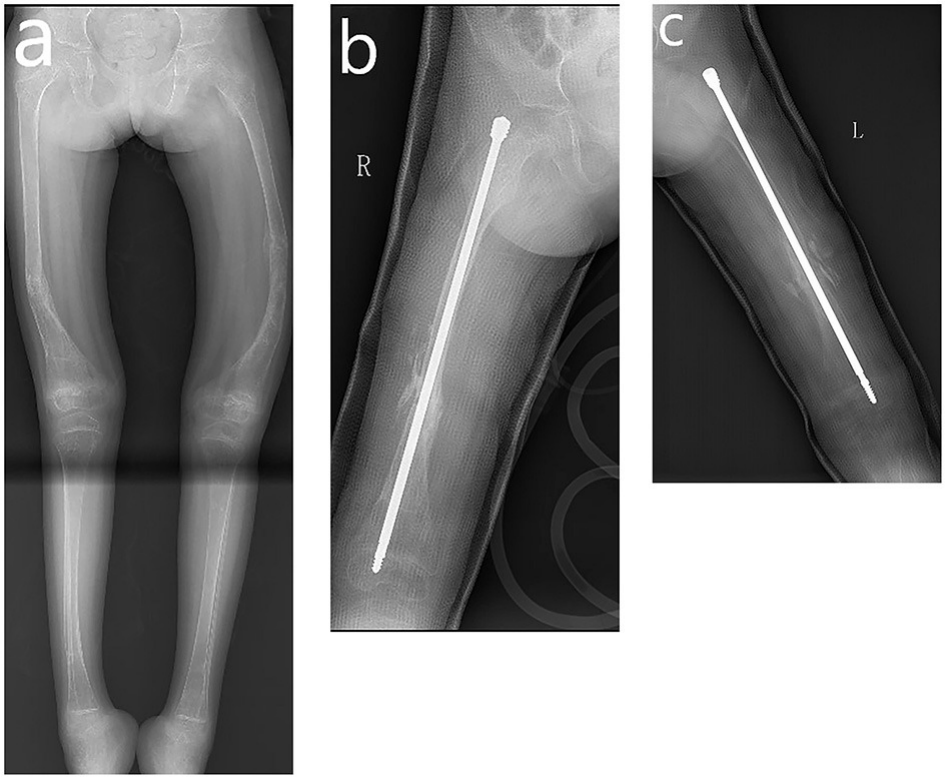
Radiograph of fractures in lower extremity. **(a)** Full-length image of the lower extremities showing fracture and bowing in bilateral femur. **(b)** Nailing of the right femur. **(c)** Nailing of the left femur.

Dual-energy X-ray absorptiometry (DXA) is the gold standard for measuring bone mineral density (BMD), making the diagnosis of osteoporosis and monitoring BMD (13). GE Lunar iDXA machine was used in our hospital to measure the BMD and monitor the effects of anti-osteoporosis therapy. During her hospital stay at our institute, DXA came up with a z-score of −5 at the lumbar spine and bilateral proximal femurs, suggesting severe osteoporosis. Multiple osteotomies and telescoping rods were used to fixate the lower extremity of this patient (see Figure 2). Because of the high rate of non-union and possible hardware failure, the cubitus varus deformity was scheduled to be corrected later. Calcium and vitamin D were orally administered to the patient immediately after surgery. Oral bisphosphonates (Alendronate Sodium Tablets, 37.5 mg once per week) were administered 1 month later after the rodding surgery.

**Figure 3:**
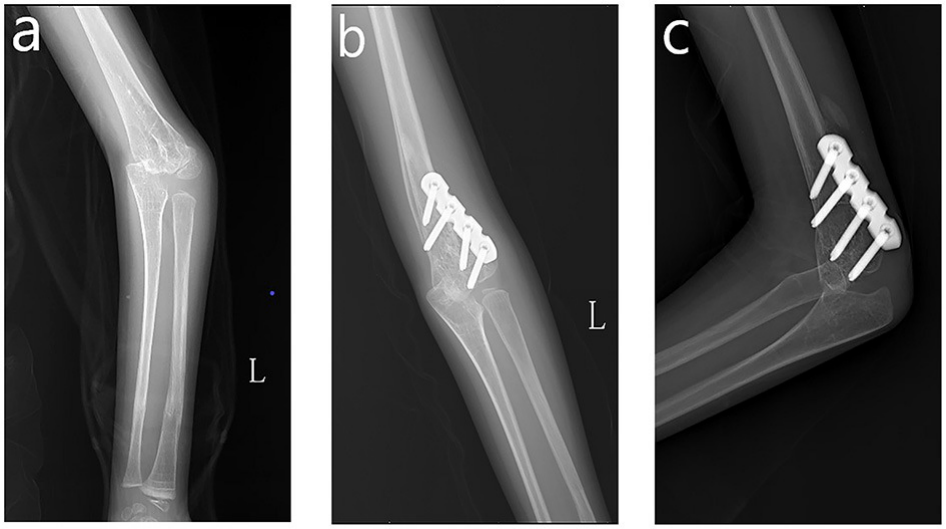
Correction of cubitus varus. **(a)** Preoperative AP view of left elbow. **(b)** AP view of the left elbow at 3-month follow-up. **(c)** Lateral view of the left elbow at 3-month follow-up.

The patient was re-admitted to our institute for cubitus varus deformity correction 8 months after the rodding surgery. The patient presented ~30 degrees of cubitus varus with prominent lateral condyle, and her contralateral elbow demonstrated ~6 degrees valgus. Our surgical plan was a simple lateral wedge osteotomy with a slight medial translation of the distal part of the humerus. In the case of poor bone quality and a significant rotational and translational rotatory force acting on the supracondylar region, Kirschner wire (KW) or external fixation does not achieve adequate mechanical stability. Therefore, a 3.5 mm locking reconstruction plate was used (see Figure 3). After the surgery, a long-arm cast was applied for 4 weeks. The oral bisphosphonates were discontinued for 3 months. The patient demonstrated full elbow motion without any pain at 1-month follow-up, and X-ray images also demonstrated early healing. At the 3-month follow-up, the patient displayed uneventful healing. The callus seemed fine and oral bisphosphonates were resumed. The DXA indicated a z-score of −3, suggesting improved BMD but still osteoporosis. The hardware was not prominent on the lateral condyle, and the plate was scheduled to be removed 12 months later. Mayo elbow performance score (MEPS) was used for the patient to assess her overall effectiveness of treatment, and the result was excellent (95 points) (14).

**Figure 4:**
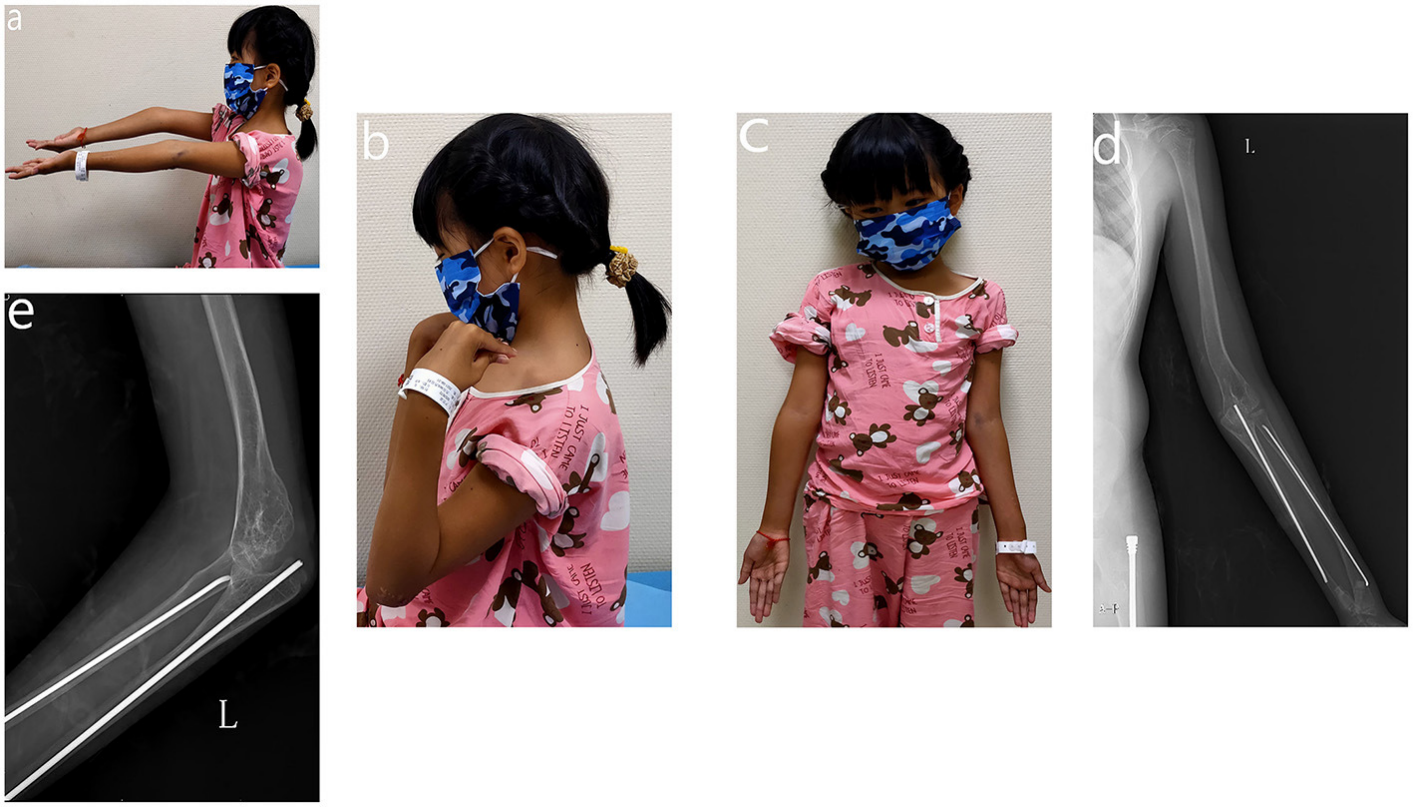
Appearance and function of 18-month after plate removal. **(a)** Extension of the elbow joint. **(b)** Flexion of the elbow joint. **(c)** Appearance of upper extremity. **(d)** AP view of the left upper extremity. **(e)** Lateral view of the left elbow joint.

Eleven months after the corrective surgery, the patient came back to our institute with the left radius fracture. An elastic stable intramedullary nail (ESIN) was used to fixate the radius and ulna and plate removal was performed at the same time. Thereafter, oral bisphosphonates (Alendronate Sodium Tablets, 37.5 mg qw) were replaced as intravenous Zoledronic acid, biannually (0.05 mg/kg). Eighteen months after plate removal, the patient received follow-up during the out-patient visit. The appearance and ROM on the left arm was satisfactory. Besides, the radiological examination also corroborated the successful correction of cubitus varus (see Figure 4). No height spurt (118 cm) or fracture was observed. The DXA indicated a z-score of −1.8, suggesting improved BMD.

All procedures performed in this study were performed in accordance with the ethical standards of the national research committee and the 1964 Helsinki declaration and its later amendments or comparable ethical standards. The patient's legal guardian provided informed consent for the publication of the case.

## Discussion

Telescoping intramedullary fixation is the standard treatment strategy for long bone fractures in OI, especially in lower extremities (15). Although telescoping rods were used for bilateral femoral deformity and fresh bilateral femoral shaft fracture in this study, they were not suitable for elbow deformity correction. There are many fixation methods regarding the correction of cubitus varus, including KW fixation, tension-band wiring, screws alone, external fixator, and plating (1, 2, 16). However, routine use of KW or external fixator might not provide enough stability in osteoporotic bone, especially in this case. Therefore, we considered a locking plate as the preferred choice for this patient.

Osteoporotic fractures represent unique fixation challenges related to decreased BMD and higher rates of screw pullout. However, Hanke et al. reported that the locking plate technique was successful as a salvage procedure in a case report of femoral fracture in OI (17). Other reports also advocate the utilization of locking plate in osteoporotic fractures and non-union of the humerus in OI (18–21). The advantage of the locking plate is the screws with angular stability, and it successfully avoids loosening of fixation due to creating a fixed-angle construct with excellent resistance to failure in osteoporotic bone with thin cortices (22). Surgical fixation of the long pathological bones of patients with OI should be intramedullary rods, which avoids the stress risers created by a stand-alone plate and screw construct (23). However, as we discussed earlier, the utilization of intramedullary rods in the supracondylar region is impossible, and KWs or external fixators in the osteoporotic bones are mechanically unstable. Therefore, a 3.5 mm locking plate was used to fixate the osteotomy site. There was a report about dual-plating, but a single lateral plate was sufficient in our case (20).

There is no gold standard surgical correction for cubitus varus deformity, and many types of osteotomies have been reported, including lateral wedge osteotomy, step-cut, dome osteotomy, and spatial correction using Taylor spatial frame (TSF) (2, 24). Considering operability and reproducibility, we chose lateral wedge osteotomy. For this patient, lateral closing wedge osteotomy combined with a slight medial translation of the distal part of the humerus was able to correct the varus deformity without residual lateral condylar prominence. As for pharmaceutical intervention, it plays an indispensable part in post-operative recovery. Bisphosphonates inhibit bone resorption by reducing the number and activity of osteoclasts: which results in improved vertebrae form and density, increased cortical diameter and increased bone volume (25). Bisphosphonate therapy has been given to children with OI for over three decades, and various studies have concluded that bisphosphonates could increase BMD in children with OI and decrease their fracture rate in the range of 30–60% (26–28). Recent studies showed that both oral and intravenous bisphosphonates therapy seemed to be associated with a lower rate of long-bone fractures in children with OI (27, 28). Trejo and Rauch suggested intravenous administration might be a better choice because it could improve mobility and positively affect the spine of growing children with OI (27). Therefore, both oral and intravenous bisphosphonates were administered to this patient.

Non-union is a known complication in patients with OI, with or without the application of bisphosphonates treatment (29, 30). Whether bisphosphonates treatment affects the healing of fractures in OI remains controversial (31, 32). However, there were reports of non-union in the humerus (19, 20, 29, 31), and non-union in the upper extremity can be disabling (33). Therefore, concerning the low BMD, we postponed the corrective surgery for more than 6 months, and calcium and vitamin D supplements were used immediately after rodding surgery to increase the patient's BMD. Subsequently, bisphosphonates were given to the patient 1 month later. However, concerning the possible impact on post-operative fracture healing, oral administration of bisphosphonates was discontinued after corrective elbow surgery and resumed after 3-month follow-up. This patient displayed an uneventful healing process without any adverse event, including non-union.

There were several limitations in our study. Firstly, intravenous bisphosphonates were not employed from the very beginning. Besides, an anatomic locking plate might be a better choice for corrective surgery if available, and cost-effective analysis using a locking plate remains to be investigated. Furthermore, updated research demonstrated that the pediatric outcomes data collection instrument could be a reliable measure of physical functioning in children with OI (34). It offers valuable information about patient-reported systemic health status. This new tool could be used in the assessment of future patients with OI.

## Conclusion

Locking plate combined with meticulous pharmacological intervention delivers satisfactory clinical results for corrective surgery of cubitus varus deformity in the pediatric patient with osteogenesis imperfecta.

## Data Availability Statement

The original contributions presented in the study are included in the article/supplementary material, further inquiries can be directed to the corresponding author/s.

## Ethics Statement

The studies involving human participants were reviewed and approved by Ethics Committee of Tongji Medical College, Huazhong University of Science and Technology. Written informed consent to participate in this study was provided by the participants' legal guardian/next of kin. Written informed consent was obtained from the individual(s), and minor(s)' legal guardian/next of kin, for the publication of any potentially identifiable images or data included in this article.

## Author Contributions

JL was in charge of the main idea and is the guarantor of integrity of the entire study. PH and RL were in charge of the study concepts, design, manuscript preparation, and editing. PH and SR were in charge of the language polishing and the grammar revision. All authors read and approved the final manuscript.

## Conflict of Interest

The authors declare that the research was conducted in the absence of any commercial or financial relationships that could be construed as a potential conflict of interest.

## Publisher's Note

All claims expressed in this article are solely those of the authors and do not necessarily represent those of their affiliated organizations, or those of the publisher, the editors and the reviewers. Any product that may be evaluated in this article, or claim that may be made by its manufacturer, is not guaranteed or endorsed by the publisher.

## Abbreviations

OI: osteogenesis imperfecta
SCHF: supracondylar humeral fracture
DXA: dual energy x-ray absorptiometry
KW: Kirschner wires
ESIN: elastic stable intramedullary nail
BMD: bone mineral density
TSF: Taylor spatial frame
MEPS: Mayo elbow performance score.
